# Why Does Insect RNA Look Degraded?

**DOI:** 10.1673/031.010.14119

**Published:** 2010-09-21

**Authors:** Eva C. Winnebeck, Craig D. Millar, Guy R. Warman

**Affiliations:** ^1^ Department of Anaesthesiology, Faculty of Medical and Health Sciences, The University of Auckland, Auckland, New Zealand; ^2^ School of Biological Sciences, Faculty of Science The University of Auckland, Auckland, New Zealand

**Keywords:** Bioanalyzer, electrophoresis, hidden break, honey bee, ribosomal RNA

## Abstract

The integrity of extracted ribonucleic acid (RNA) is commonly assessed by gel electrophoresis and subsequent analysis of the ribosomal RNA (rRNA) bands. Using the honey bee, *Apis mellifera* (Hymenoptera: Apidae), as an example, the electrophoretic rRNA profile of insects is explained. This profile differs significantly from the standard benchmark since the 28S rRNA of most insects contains an endogenous “hidden break.” Upon denaturation, the masking hydrogen bonds are disrupted, releasing two similar sized fragments that both migrate closely with 18S rRNA. The resulting rRNA profile thus reflects the endogenous composition of insect rRNA and should not be misinterpreted as degradation.

## Introduction

Gene expression studies employing methods such as RT-PCR, quantitative RT-PCR, and microarrays are now routinely conducted in many areas of molecular biology. An essential prerequisite for these studies is successful extraction of the ribonucleic acids (RNA). Extracting RNA is generally complicated by its high lability caused by presence of ribonucleases (RNases) within the sample as well as in the environment. For this reason, RNA degradation can be a significant issue, and thus the integrity of the extracted RNA is usually determined before its application in down-stream procedures.

Gel electrophoresis and inspection of the prominent bands of the highly abundant ribosomal RNAs (rRNAs) is the most common method for assessing the integrity of extracted RNA. Analyzed in particular are the large rRNAs, designated 16S and 23S in prokaryotes and 18S and 28S in eukaryotes. An intact sample is generally defined as displaying clear and distinct bands for both these large rRNA species. This is based on the assumption that rRNA integrity reflects the integrity of the other fractions of RNA, for example the mRNA fraction in the case of expression studies. While rRNA integrity may not necessarily be an accurate measure of mRNA quality, it is certainly useful as a readily available indicator of the general state of the purified RNA.

The rRNA profile of the honey bee, *Apis mellifera* (Hymenoptera: Apidae), as obtained via electrophoresis with the Agilent 2100 Bioanalyzer, is reported here. Using these results as an example, the electrophoretic rRNA profile of insects in general is detailed. A reminder and a short review explaining why this profile differs significantly from the standard rRNA integrity benchmark is provided.

## Materials and Methods

*A. mellifera* RNA was obtained from brains of forager bees collected into liquid nitrogen. The brains were dissected frozen on a precooled aluminium block (-80° C) surrounded by dry ice. Single brains were then homogenized in 320 µl RLT lysis buffer (QIAGEN RNeasy Mini Kit, www.qiagen.com) using a rotor-stator homogenizer. Subsequently, total RNA was extracted via silica-matrix spin columns (QIAGEN RNeasy Mini Kit) including an on-column DNase I treatment (QIAGEN DNase I, RNase-free), both according to the manufacturer's instructions. For quality and integrity analysis, *A. mellifera* brain RNA (70 ng) was electrophoretically separated with an Agilent 2100 Bioanalyzer using an RNA 6000 Nano Chip Kit. When RNA was heat-denatured prior to separation (as recommended), RNA was incubated at 70° C for 2 min.

## Results and Discussion

In a study of gene expression in *A. mellifera,* extracted RNA was analysed for integrity via gel electrophoresis. To this end, an Agilent 2100 Bioanalyzer was used. This instrument is one of the recent microfluidic capillary electrophoresis systems that have become the current gold standard for RNA quality assessment. Separation is based on a non-denaturing gel matrix; however, samples are usually heat-denatured prior to separation (Krupp 2005).

**Figure 1.**
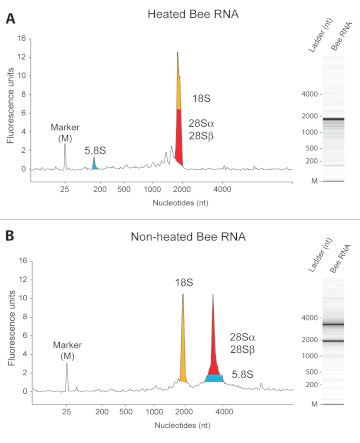
Electrophoretic profiles and virtual gels *of Apis mellifera* RNA. (A) *A. mellifera* brain RNA profile after heat-denaturation of two minutes at 70° C. (B) *A. mellifera* brain RNA profile without prior heat-denaturation. The main constituents of peaks are given: yellow denotes 18S; blue, 5.8S; and red, α and β fragments of 28S rRNA. Colored areas under the curve are only illustrative and not quantitative. High quality figures are available online.

The *A. mellifera* rRNA profiles were surprising: they consistently showed a single rRNA peak instead of two clear peaks expected for the two large rRNA species, 18S and 28S (Figure 1A). Discussion with other insect researchers suggested that a single rRNA peak is commonly seen in insect RNA and probably does not represent degradation, although none could provide an explanation for this phenomenon.

Interestingly, when samples were not heat-denatured, the expected two 18S and 28S rRNA peaks (Figure 1B) were observed. It seemed that heating converted the 28S rRNA into fragments that reappeared at least partly with the 18S rRNA fraction. This result could also be seen on standard non-denaturing agarose gels (data not shown). A detailed literature survey demonstrated that this 28S rRNA thermolability in insects had been investigated in the rRNA field 30–40 years ago. However, it appears that today, few molecular biologists are aware of these findings, resulting in a common and unfortunate misinterpretation of many insect RNA profiles.

**Figure 2.**
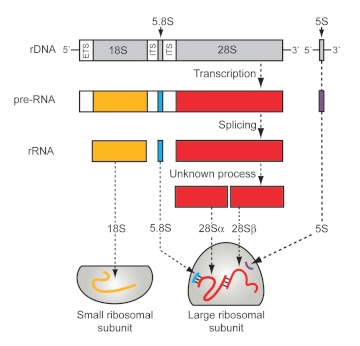
Assembly of rRNA into the ribosomal subunits in insects. In eukaryotes, each cytoplasmic ribosome comprises four different molecules of RNA named after their approximate sedimentation properties: two large rRNAs designated 18S and 28S and two small rRNAs termed 5S and 5.8S. The 18S rRNA composes the major part of the small ribosomal subunit, whereas the other three rRNAs constitute the RNA component of the large ribosomal subunit. While all four rRNAs of cytoplasmic ribosomes are encoded in nuclear genes, the 18S, 5.8S, and 28S rRNAs are transcribed as a single large precursor molecule. Post-transcriptional processing of this pre-rRNA in the nucleolus subsequently yields separate mature rRNAs that assemble with numerous ribosomal proteins to form the ribosomal subunits. Most insects show an additional processing step that cleaves the 28S rRNA into α and β fragments, which remain hydrogen-bonded together. Colors refer to the color-coding in Figure 1. ETS denotes the externally transcribed spacer region; ITS stands for the internally transcribed spacer regions. For simplicity, the extensive secondary structure of the rRNAs is not reflected in the diagram. Modified from Gillespie et al. (2006). High quality figures are available online.

Reports of the heat-induced breakdown of insect 28S rRNA accumulated in the 1960s and ′70s, and the phenomenon was first characterised in Lepidoptera and Diptera (Applebaum et al. 1966; Greenberg 1969; Rubinstein and Clever 1971; Ishikawa and Newburgh 1972). It was found that, after heating the RNA to 40–60° C for a few minutes, the 28S rRNA fraction of these taxa suddenly sedimented alongside the 18S rRNA fraction, while the latter was unaffected. This rapid thermoconversion of 28 S rRNA occurred without detectable intermediates and was highly reproducible. It was therefore attributed to a specific scission near the centre of the molecule that resulted in separation into two fragments of similar length, later designated α and β. Although incidentally migrating with 18S rRNA, the 28S fragments were demonstrated to be distinct from 18S rRNA as the difference in base composition between the 28S and 18S rRNA was maintained even after heat-fragmentation (Ishikawa and Newburgh 1972). This thermolability did not appear to be an artifact of extraction since RNase inhibitors were ineffective in preventing the breakdown (Applebaum et al. 1966; Rubinstein and Clever 1971), and rRNA (co-) extracted from other organisms such as *Escherichia coli,* rat, or hamster did not display any such rapid heat-induced fragmentation (Applebaum et al. 1966; Shine and Dalgarno 1973). If not introduced during extraction, the scission had to be an intrinsic feature of the 28S rRNA of these insects, a pre-existing “hidden break” (coined by Ishikawa and Newburgh (1972) after Gould (1967)) in the RNA backbone. This hidden break was suggested to be introduced into the polynucleotide chain rather late in the maturation of the 28 S rRNA, following pulse-chase experiments which demonstrated that the immediate precursors did not heat-dissociate into two major fragments, in contrast to its mature 28S product (Applebaum et al. 1966; Ishikawa and Newburgh 1972). Analyses of fragmentation conditions suggested that non-covalent RNA interactions, mainly hydrogen bonds, were most probably holding the 28S rRNA together, thereby successfully “hiding the break” (Figure 2).

It quickly became clear that the occurrence of a central hidden break in 28S rRNA is not restricted to Diptera and Lepidoptera. Numerous other insects including *A. mellifera* (Hymenoptera) (Shine and Dalgarno 1973; De Lucca et al. 1974; Gillespie et al. 2006) display the phenomenon of 28S thermolability. In fact, the only insects found not to have fragmented 28S rRNA are the aphids (Shine and Dalgarno 1973; Ishikawa 1977; Basile-Borgia et al. 2005). Furthermore, scissions in the 28S rRNA extend even beyond the class of insects and have been shown not only in other arthropods, but also many other protostomes (Ishikawa 1977). Surprisingly, they have even been discovered in mammals, namely in several South American rodent species of the genus *Ctenomys* (Melen et al. 1999).

Research over the past 40 years has shed more light on the nature of hidden breaks in rRNA, while their biological significance still remains an enigma (Basile-Borgia et al. 2005). The central hidden break in 28S rRNA was found to result not from a single scission, but from a double cleavage event with excision of the middle fragment; the length of this released fragment varies with the species in question. Several cleavage signals have been proposed, mainly located within AU-rich regions, but no consensus sequence has yet been identified. Concordantly, the processing machinery of the excision is still entirely unknown. The location of the central scission site in insects was identified in one of the highly variable regions, expansion segment D7a (Ware et al. 1985), which also constitutes the probable site of fragmentation in *A. mellifera* (Gillespie et al. 2006). Therefore, in our case, the 28S fragments in the honey bee are approximately 1900 (α) and 2000 (β) nucleotides in length. As the 18S rRNA is of similar size (1923 nt) (Gillespie et al. 2006), these three molecules appear as a single peak after denaturation (Figure 1A).

Upon heating the *A. mellifera* RNA, a new peak of approximately 160 nucleotides in size (Figure 1A) also became apparent. In bees, this was first reported by De Lucca et al. (De Lucca et al. 1974); however, it is neither specific for bees nor for insects in general. The small component released through denaturation also originates from the 28S rRNA complex, although it is not another product of internal cleavage within an rRNA species, but rather a separate rRNA: 5.8S rRNA. First demonstrated by Pene et al. (1968) and King and Gould (1970), 5.8S rRNA is base-paired to the 28S rRNA in all eukaryotic cytoplasmic ribosomes (Figure 2) and released upon denaturation (Figures 1A, 1B).

It is interesting to note that since the 5.8S rRNA is hydrogen bonded to the 28S rRNA, it generally co-purifies with the large rRNA species. Accordingly, when extracting RNA using silica-matrix spin columns such as the Qiagen RNeasy kits, 5.8S rRNA is purified despite the exclusion of RNA below 200 nucleotides in length. This is contrary to statements in application notes by Agilent and Qiagen (e.g. Krupp 2005; Massotti and Preckel 2006; Qiagen 2006).

## Conclusion

In conclusion, the 28S rRNA of most insects consists of two separate fragments that are hydrogen-bonded together. Depending on pretreatment and electrophoresis conditions (native or denaturing), disruption of these hydrogen bonds occurs, and the two fragments co-migrate with the 18S rRNA. The 5.8S rRNA is also base-paired to this 28S complex and is likewise released in denaturing conditions. Therefore, the typically observed insect rRNA profile reflects endogenously present components of the insect rRNA rather than degradation during the extraction process. A lack of awareness of the rRNA composition in insects has led to poor interpretation of insect rRNA profiles both in daily experiments as well as in publications.
